# 

**Published:** 1874-07

**Authors:** 


ESTABLISHED IN 1855.

VOLUME XII. | JULY—1874. | NUMBER 4.

### ATLANTA

### Medical and Surgical Journal.

MONTHLY: THREE DOLLARS PER ANNUM, IN ADVANCE.

ROBERT BATTEY, M.D.,
EDITOR.

WM. ABRAM LOVE, M.D., V. H. TALIAFERRO, M.D.,
ASSOCIATE EDITORS.

*PAX ET SCIENTIA, SED VERITAS SINE TIMORE.*

ATLANTA, GEORGIA:
*DUNLOP, WYNNE & CO., BOOK AND JOB PRINTERS.*
1874.

